# A decade of global orthopaedic research in *SICOT-J* (2015–2025): a scientometric analysis of publication trends, collaboration, and citation impact

**DOI:** 10.1051/sicotj/2026021

**Published:** 2026-05-25

**Authors:** Raju Vaishya, Brij Mohan Gupta, Mallikarjun Kappi, Abhishek Vaish, Andreas F Mavrogenis, Sébastien Lustig, Vikas Khanduja

**Affiliations:** 1 Department of Orthopaedics and Joint Replacement Surgery, Indraprastha Apollo Hospitals, Sarita Vihar New Delhi 110076 India; 2 CSIR-NISTADS, Pusa New Delhi – 110012 India; 3 Library and Information Centre, Government First Grade College Hosapete 583201 Karnataka India; 4 First Department of Orthopaedics, National and Kapodistrian University of Athens School of Medicine 15562 Athens Greece; 5 Hôpital de la Croix Rousse, Université Claude Bernard Lyon I France; 6 Young Adult Hip Service, Department of Trauma & Orthopaedics, Addenbrooke’s – Cambridge University Hospital Cambridge UK

**Keywords:** Bibliometrics, Orthopaedic, Research Collaboration, Publication Trends, Trauma

## Abstract

*Background*: The *SICOT-J*, an open-access orthopedic journal affiliated with the Société Internationale de Chirurgie Orthopédique et de Traumatologie (SICOT), has been a global orthopedic research platform since 2015. This study provides a scientometric analysis of *SICOT-J* publications from 2015 to 2025 to evaluate research productivity, citation impact, and collaboration patterns. *Methods*: 542 documents published in *SICOT-J* and indexed in the Scopus database were analyzed using bibliometric indicators. Data on publication types, subject areas, authorship, institutional and geographic affiliations, funding disclosures, collaboration networks, and citation metrics were extracted. Microsoft Excel was used for data processing and analysis. Key indicators included citations per publication (CPP), relative citation index (RCI), and total link strength (TLS) for collaborative connections. *Results*: From January 1 2015, to June 30 2025, *SICOT-J* published 542 articles with an overall CPP of 10.29. Research articles comprised 78.6% of publications, while reviews – though fewer in number – had the highest CPP (23.38). The most frequent topics were arthroplasty (25.83%) and trauma/fractures (24.17%), with the hip and knee as the most studied anatomical regions. Only 5.17% of the articles reported external funding. Fifteen highly cited papers (≥50 citations) were mostly reviews and internationally co-authored. *Conclusions*: *SICOT-J* has demonstrated consistent publication growth and international participation, though with notable concentration in a few high-income countries. The high citation impact of reviews and collaborative works highlights the importance of strategic content development. Enhancing funding transparency, supporting underrepresented regions, and promoting emerging topics strengthen the journal’s global impact.

## Introduction

The evolution of orthopedic research has been significantly shaped by global scholarly output, with speciality journals as critical platforms for disseminating clinical and scientific advancements. *SICOT-J* (https://www.sicot-j.org/), the official open-access journal of the Société Internationale de Chirurgie Orthopédique et de Traumatologie (SICOT), has emerged as a reputable forum for orthopedic scholarship since its inception in 2015. As orthopedic surgery continues to evolve rapidly – with growing emphasis on arthroplasty, trauma management, minimally invasive procedures, regenerative medicine, and biomechanics – quantitative evaluations of research dissemination have become increasingly essential to guide policy, funding, and clinical practice priorities [1].

Scientometric and bibliometric analyses have become widely adopted tools for evaluating research output, collaboration, and impact across medical fields [2, 3]. Bibliometric methods are valuable for assessing longitudinal publication trends, institutional productivity, international cooperation, and citation influence within specific disciplines [4]. In orthopedics, prior bibliometric studies have examined top-cited articles on several themes [5–11], providing crucial insights into global trends. However, journal-specific scientometric evaluations remain limited [12–14]. Bibliometric analyses tailored to individual journals can reveal granular insights into regional contributions, high-impact authors, and institutional collaboration networks. Moreover, they offer transparency into underrepresented topics or geographies and inform editorial and strategic decisions [15, 16].

This study presents a detailed scientometric analysis of *SICOT-J* publications from 2015 to 2025, aiming to assess its publication growth, citation performance, thematic and geographic distribution, and collaborative linkages. By analysing 542 Scopus-indexed publications, the study offers an evidence-based appraisal of the journal’s global scientific influence and provides data to guide future orthopedic research directions.

## Materials and methods

The global orthopedic documents covered in *SICOT-J* and indexed in the Scopus database, spanning 11 years from January 1, 2015, to June 30, 2025, were analyzed using bibliometric methods in this study. To identify and download all documents indexed in *SICOT-J,* a search strategy was developed that used *SICOT-J* as a source tag and restricted the publication years to 2015-2025, which initially yielded 542 global documents. The search strategy used was: “SRCTITLE (SICOT-J)”.

For each publication record downloaded, the information collected includes author and institution details, funding, collaboration, citations, and publication type/source, all analyzed using MS Excel for data cleaning, preliminary organization, and descriptive statistics; Biblioshny, the web interface of the Bibliometrix R-package, to generate bibliometric indicators and thematic structures; and VOS viewer for constructing and visualizing co-authorship and citation networks. The bibliometric analysis focused on several key indicators: assessing overall publication trends and growth, characterizing publication types and sources, evaluating the extent of external funding and identifying supporting agencies, determining the scope of international collaboration by identifying key countries, organizations, and authors, and analyzing the geographical distribution of publications. Additionally, the study classified papers by broad subject areas, keywords, and organ/bone focus, and identified the top 22 most productive organizations (with ≥13 papers), the top 19 most productive authors (with ≥13 papers), and the top 15 highly cited papers or HCPs (with ≥50 citations).

## Results

### Annual and cumulative growth of publications in *SICOT-J*

**Figure 1:**
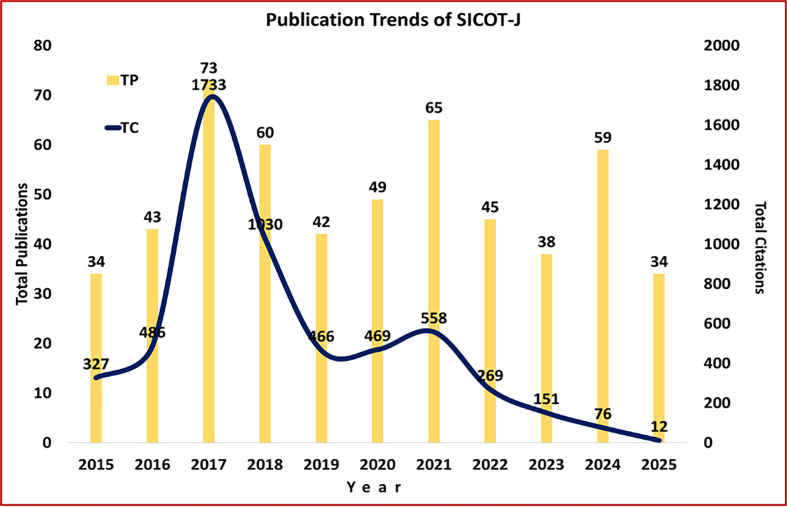
Publication and citation trends of *SICOT-J* from January 1, 2015, to June 30, 2025.

The *SICOT-J* witnessed moderate growth in the annual publications, increasing from 34 in 2015 to 59 in 2024, reaching peaks of 73 and 65 in 2016 and 2021 (Figure 1). The total publications (TP), 508, registered an annual average growth rate of 11.85%. However, the 5-year cumulative publications marginally increased from 252 to 256, registering 1.59% absolute growth. Over these 10 years, the journal published 508 articles, achieving 5565 total citations (TC), yielding an overall average of 10.95 citations per publication (CPP). However, the 5-year cumulative citation impact per paper declined from 16.4 during 2015–2019 to 5.95 during 2015–2024 (Table 1). It should be noted, though, that the decline in citation impact is not real, as the publications in the later period did not get enough time to gain citations (“citation lag”).

**Table 1:** Annual growth of publications in the SICOT-J (2015–2025).

Total publications			
Year	TP	TC	CPP
2015	34	327	9.62
2016	43	486	11.30
2017	73	1733	23.74

Research articles were the most common publications (78.60%), followed by the review articles (18.63%). However, review articles received much higher average citations per paper (CPP-23.38), as compared to the research articles (CPP-7.51) (Supplementary Table 1).

Keyword subject analysis reveals that “Arthroplasty” and “Arthroscopy” are the most frequently occurring keywords in 18 papers each (Supplementary Figure 1). This is closely followed by “Osteoarthritis” (11 papers), “Intermedullary Nails (9 papers), “Fractures”, “Rehabilitation” and “Hemiarthroplasty” (7 papers each), “Infection” and Dislocation” (6 papers each), “Trauma”, “Debridement” and Biomechanics” (5 papers each), “Sarcoma”, “Reconstruction”, Prosthetic Joint Infection” and Covid-19 (4 papers each), Osteotomy (3 papers) and Giant Cell Tumor (2 papers) (Supplementary Table 2).

### Top countries

Sixty-five countries contributed to *SICOT-J* publications, with the top 10 nations producing 533 papers and 5,809 citations, accounting for 98.34% of all publications and over 100% of total citations due to international co-authorships. France led with 95 papers, followed by the United Kingdom (UK) (*n* = 86), Egypt (*n* = 79), and both India and the United States (US) (*n* = 54 each). Five countries – Australia, the US, the UK, Germany, and Italy – exceeded the average citation impact (CPP >10.90, RCI >1.06), with Australia achieving the highest CPP (20.09) and RCI (1.95).

France also exhibited the strongest research collaboration with the highest Total Link Strength (TLS) of 154, followed by the UK (76) and Egypt (59). The most frequent bilateral collaboration was between the UK and Egypt (14 linkages), with strong ties also seen between France and Greece (12), and France with both Italy and Australia (10 each), highlighting a robust network of international orthopedic research partnerships. The countries’ collaboration network is divided into three clusters based on co-authorship and citation metrics (Supplementary Figure 2).

The bibliometric details of the top 10 countries publishing in the SICOT-J are presented in Table 2.

**Table 2:** Top 10 Countries publishing in SICOT-J (2015–2025).

S. No	Name of the country	TP	TC	CPP	RCI	TLS	Collaborating countries (links)
1	France	95	852	8.97	0.87	154	2(6), 3(6), 4(2), 5(3), 7(12), 8(10), 10(10)
2	United Kingdom	86	1195	13.90	1.35	76	1(6), 3(14), 4(5), 5(5), 6(1), 7(4). 8(2), 10(1)
3	Egypt	79	721	9.13	0.89	59	1(2), 2(14), 4(3), 5(4), 6(2), 8(2), 9(6)
4	India	54	395	7.31	0.71	23	1(2), 2(5), 3(3), 5(2), 10(1)
5	United States	54	1007	18.65	1.81	51	1(3). 2(5), 3(4), 4(2), 7(1), 8(1), 9(2), 10(3)
6	Japan	47	361	7.68	0.75	20	2(1), 3(1), 7(5), 8(5)
7	Greece	40	210	5.25	0.51	44	1(12), 2(4), 5(1), 6(5), 8(9), 9(4), 10(2)
8	Italy	32	331	10.34	1.01	46	1(10), 2(2), 3(2), 5(1), 6(5), 7(9), 9(1)
9	Germany	23	275	11.96	1.16	16	3(6), 5(2), 7(4), 8(1)
10	Australia	23	462	20.09	1.95	14	1(10), 2(2), 4(1), 5(3), 7(2)
		533	5809	10.90	1.06	503	
		542	5577	10.29	1.00		
		98.34					

TP: Total Publications; TC: Total Citations; CPP: Citations Per Publication; RCI: Relative Citation Index; TLS: Total Link Strength.

### Top organizations

A total of 313 organisations contributed to publications in *SICOT-J* between 2015 and 2025, with the top 22 organisations producing 455 papers (83.95% of total output) and receiving 4,452 citations (79.83% of total citations) (Supplementary Table 3).

Nine organisations surpassed the average productivity threshold (≥20.68 papers), led by Université Claude Bernard Lyon 1 (42 papers), Université de Lyon (41), and Hôpital de la Croix-Rousse (40). In terms of citation impact, nine institutions exceeded the average CPP of 9.78 and RCI of 0.95, with Aarhus Universitetshospital, Denmark (CPP 16.64; RCI 1.62) and Addenbrooke’s Hospital, UK (CPP 15.75; RCI 1.53) being the most impactful (Table 3). These findings highlight the strong contribution and influence of a small group of predominantly European institutions in shaping the orthopedic research landscape within *SICOT-J*.

**Table 3:** Bibliometric profile of the top six impactful organizations.

S.No	Name of the organization	TP	TC	CPP	RCI
1	Aarhus Universitetshospital, Denmark	11	183	16.64	1.62
2	Addenbrooke’s Hospital, Cambridge, UK	8	126	15.75	1.53
3	Laboratoire de Biomécanique et Mécanique des Chocs, France	36	404	11.22	1.09
4	Hôpital de la Croix-Rousse, France	40	427	10.68	1.04
5	Université Gustave Eiffel, France	37	391	10.57	1.03
6	Université Claude Bernard Lyon 1, France	42	435	10.36	1.01

TP: Total Publications; TC: Total Citations; CPP: Citations Per Publication; RCI: Relative Citation Index.

The TLS, indicating research collaboration intensity among the top 22 contributing organisations in *SICOT-J*, ranged from 13 to 328. The highest TLS was recorded by Université de Lyon, France (328), closely followed by Université Claude Bernard Lyon 1 (327), and Hôpital de la Croix-Rousse (297), all highlighting France’s dominant collaborative presence. Other major French institutions, such as the Fédération Internationale de Football Association (286), Université Gustave Eiffel (284), and Laboratoire de Biomécanique et Mécanique des Chocs (284), also showed strong network centrality. Outside France, notable collaboration metrics were observed in the National and Kapodistrian University of Athens (69 TLS), Cairo University (43), and Addenbrooke’s Hospital, UK (38). The most frequent bilateral collaborations occurred between Université Claude Bernard Lyon 1 and Université de Lyon (38 linkages), followed by partnerships involving Claude Bernard Lyon 1 with Hôpital de la Croix-Rousse, Université Gustave Eiffel, and Laboratoire de Biomécanique (36 linkages each), reflecting a tightly interconnected French research network (Supplementary Figure 3).

### Top authors

A total of 1,893 authors contributed to *SICOT-J* between 2015 and 2025, with the top 19 most productive authors collectively publishing 256 papers and receiving 2,875 citations, accounting for 47.23% of total publications and 51.55% of total citations (Supplementary Table 4). E. Sappey-Marinier (France) ranked as the most impactful author with a CPP of 32.44 and RCI of 3.15. At the same time, other high-impact contributors included V. Khanduja (UK), K. Kaneko (Japan), and J. Shatrov (France) (Table 4). The average international collaboration rate among these leading authors was 50.78%, with wide variability ranging from no collaboration to full international participation.

**Table 4:** Bibliometric profile of the top six impactful authors.

S.No	Name of the author	Affiliation of the author	TP	TC	CPP	RCI
1	E. Sappey – Marinier	Hôpital de la Croix-Rousse, France	9	292	32.44	3.15
2	V. Khanduja	Addenbrooke’s Hospital, Cambridge, UK	7	102	14.57	1.42
3	K. Kaneko	Juntendo University School of Medicine, Japan	12	157	13.08	1.27
4	J. Shatrov	Hôpital de la Croix-Rousse, France	8	104	13.00	1.26
5	Y. Homma	Addenbrooke’s Hospital, Cambridge, UK	10	127	12.70	1.23
6	E. Servien	Hôpital de la Croix-Rousse, France	35	418	11.94	1.16

TP: Total Publications; TC: Total Citations; CPP: Citations Per Publication; RCI: Relative Citation Index.

Among the top 19 most productive authors in *SICOT-J*, TLS ranged from 12 to 234. S. Lustig (France) exhibited the highest TLS (234), followed by E. Servien (190), C. Batailler (169), A.F. Mavrogenis (106), and T. Baba (75), indicating strong collaborative influence. The number of bilateral collaborative linkages among these authors ranged from 1 to 35, with the strongest partnership observed between S. Lustig and E. Servien (35 collaborations). Other prominent author pairs included Lustig–Batailler (32 linkages), Servien–Batailler (29), and Lustig–Sappey-Marinier (9), underscoring a tightly interconnected core group of French researchers driving a significant portion of the journal’s collaborative output (Supplementary Figure 4).

### Highly-Cited Papers (HCPs)

Between 2015 and 2025, *SICOT-J* published 542 articles, of which only 15 (2.77%) qualified as HCPs, each receiving 50 or more citations. These HCPs were published between 2017 and 2022, collectively garnered 1,473 citations, averaging 98.2 CPP. Most of these papers (11) fell within the 51–88 citation range, while the remaining four achieved between 100 and 395 citations. The majority were published in 2017 (10 papers) and 2021 (2 papers), and the collection included 12 reviews and 3 original research articles, highlighting the higher citation impact of review publications. Collaboration was prominent in 10 of these papers (3 national and 7 international), with the US and UK involved in four each, and Australia and France in three; only five papers (33.3%) were authored by single institutions. Contributions came from 59 authors and 41 institutions, with top-producing institutions including Hôpital de la Croix-Rousse, Université Claude Bernard Lyon 1, Université de Lyon (France), and Chandigarh (India) – each involved in two HCPs that collectively received 213 citations. S. Lustig and E. Servien were the leading contributors, each co-authoring two of the top-cited papers. Full bibliographic details of the top 15 HCPs are provided in Table 5.

**Table 5:** Bibliometric profile of the top 15 Highly Cited Papers of *SICOT-J.*

S.No	Names of authors	Title and source	Citations
1	Misaghi, A., Goldin, A., et al.	Osteosarcoma: A comprehensive review. *SICOT-J.* 2018: 4: 12.	395
2	Qasim, S.N., Swann, A., Ashford, R.	The DAIR (debridement, antibiotics, and implant retention) procedure for infected total knee replacement – A literature review. *SICOT-J*. 2017;3:2.	141
3	Lustig, S., Sappey-Marinier, E., et al.	Personalized alignment in total knee arthroplasty: Current concepts. *SICOT-J*. 2021;7:19.	123
4	Tetsworth, K., Block, S., Glatt, V.	Putting 3D modelling and 3D printing into practice: Virtual surgery and preoperative planning to reconstruct complex post-traumatic skeletal deformities and defects. *SICOT-J*. 2017;3:16.	100
5	Mavrogenis, A.F., Igoumenou, V.G., et al.	Giant cell tumor of bone revisited. *SICOT-J*. 2017;3:54.	88
6	Shatrov, J., Battelier, C., et al.	Functional Alignment Philosophy in Total Knee Arthroplasty â ?? Rationale and technique for the varus morphotype using a CT based robotic platform and individualized planning. *SICOT-J*. 2022;8:11.	80
7	Liow, M.H.L., Chin, P.L., et al.	THINK surgical TSolution-One®(Robodoc) total knee arthroplasty. *SICOT-J*. 2017;3:63.	74
8	Hussain, N., Johal, H., Bhandari, M.	An evidence-based evaluation on the use of platelet rich plasma in orthopaedics – a review of the literature. *SICOT-J*. 2017;3:57.	67
9	Fontalis, A., Epinette, J.-A., et al.	Advances and innovations in total hip arthroplasty. *SICOT-J*. 2021;7:26.	62
10	Baer, M., Neuhaus, V., et al.	Influence of mobilization and weight bearing on in-hospital outcome in geriatric patients with hip fractures. *SICOT-J*. 2019;5:4.	62
11	Deep, K., Shankar, S., Mahendra, A.	Computer assisted navigation in total Knee and hip arthroplasty. *SICOT-J*. 2017;3:50.	62
12	De Bruycker, M., Verdonk, P.C.M., Verdonk, R.C.	Meniscal allograft transplantation: A meta-analysis. *SICOT-J*. 2017;3:33.	58
13	Imam, M.A., Mahmoud, S.S.S. et al.	A systematic review of the concept and clinical applications of Bone Marrow Aspirate Concentrate in Orthopaedics. *SICOT-J*. 2017;3:17.	57
14	Dhillon, M.S., Patel, S., John, R.	PRP in OA knee – Update, current confusions and future options. *SICOT-J*. 2017;3:27.	53
15	Burt, A.M., Huang, B.K.	Imaging review of lipomatous musculoskeletal lesions. *SICOT-J*. 2017;3:34.	51

## Discussion

This scientometric analysis provides a comprehensive overview of *SICOT-J*’s publication and citation performance over the last decade (2015–2025), examining 542 documents indexed in Scopus. The findings indicate a moderately growing publication trajectory, a diverse thematic and geographic distribution, and evolving trends in orthopedic research focus, authorship, collaboration, and citation impact. The average CPP of *SICOT-J* publications was 10.29. Research Articles constituted the majority of publications (78.6%), with reviews representing a smaller share (18.6%) but demonstrating the highest citation impact (CPP: 23.38). The most productive subjects included arthroplasty (25.83%) and trauma (24.17%), aligning with broader orthopedic priorities. Regionally, hip and knee topics dominated publication frequency and citation impact. Prominent contributors included authors from France, the UK, Egypt, India, and the US. Only 5.17% of papers acknowledged external funding, and a small proportion (2.77%) of the articles qualified as HCPs with ≥50 citations.

The results of this study align with previously published bibliometric research in orthopedics [8–10, 12], which consistently report that review articles and collaborative publications accrue higher citations than individual or non-review studies. Additionally, reviews comprise a minority of orthopedic publications but dominate citation count due to their summative nature and utility for clinical practice and policy development [17, 18]. Studies have revealed a growing trend in international collaboration and emphasized the substantial citation impact of co-authored papers [5, 19]. The dominance of arthroplasty and trauma as thematic areas is consistent with other journal-focused analyses. Hip and Knee arthroplasty, trauma, and sports injuries were found to be the most frequent research areas in East Asian orthopedic journals, mirroring the focus in *SICOT-J* [5, 19]. These findings reflect these conditions’ high clinical and economic burden globally and the ongoing need for innovation and evidence-based management [20].

The low rate of funded studies (5.17%) is lower than those reported in broader biomedical fields, where funding disclosures often range from 15% to 30% depending on region and field [21]. This may point to a relative underinvestment in the orthopedic research published in *SICOT-J*, or underreporting of funding sources. The international distribution of contributors – dominated by high-income countries (HICs) – follows known patterns in orthopedic literature [14]. According to Sweileh et al., low- and middle-income countries (LMICs) are often underrepresented in orthopedic research due to limited research infrastructure and funding [3, 15]. Nonetheless, *SICOT-J* demonstrates commendable international engagement, with contributions from 65 countries and productive hubs in Egypt and India, suggesting a degree of openness and global inclusivity.

Identifying HCPs concentrated between 2017 and 2022 also parallels trends in citation dynamics. As noted by Hirsch and confirmed in several citation analyses, papers typically receive the majority of their citations within the first 3–5 years of publication [21]. Most HCPs in *SICOT-J* were reviews, often involving international collaboration – another known predictor of higher citation impact [22].

With contributions from over 65 countries, *SICOT-J* promotes global collaboration and accessibility, reflecting the society’s mission to improve orthopedic care worldwide. The journal is indexed in major databases, including Scopus, PubMed Central, and Web of Science, and has steadily gained recognition through increasing citation metrics and international authorship. Over the past decade, *SICOT-J* has steadily increased its academic footprint, reflected in several journal-level metrics. According to Clarivate’s Journal Citation Reports (2025), the journal’s 2024 Journal Impact Factor (JIF) and 5-year JIF stand at 2.3. In Scopus, the CiteScore has risen from 1.7 in 2020 to 3.4 in 2024 (Supplementary Figure 5), ranking *159/568* in surgery and *127/332* in Orthopadics & Sports Medicine (Q2) (https://www.sicot-j.org/). These metrics collectively indicate that *SICOT-J* has evolved from a niche open-access title into a well-regarded venue for orthopedic scholarship, earning improved visibility, citation influence, and global reach over time.

### Study’s limitations

Despite its strengths, several limitations must be acknowledged. First, the study relies solely on Scopus-indexed data, potentially omitting relevant papers not captured in that database. Although Scopus offers wide coverage, some discrepancies in indexing practices may affect completeness. Second, the citation analysis may be skewed for recent publications (2023–2025) due to the time lag in citation accrual. As noted by Bornmann and Daniel, citation metrics are temporally biased, and newer papers inherently have fewer citations [23]. Another limitation is the exclusive focus on quantitative indicators without a qualitative content assessment. Citation counts do not always reflect scientific quality or clinical relevance [24]. As our analysis relied on Scopus metadata, which classifies articles broadly as “Review,” it was not possible to reliably differentiate between subtypes such as systematic reviews, meta-analyses, and narrative reviews. Moreover, the study does not assess altmetrics or social media influence, which are increasingly used to gauge research dissemination and impact beyond academia [25]. Finally, the low reporting of funding information may reflect actual funding scarcity or underreporting, limiting the ability to correlate funding with research impact comprehensively.

To enhance the visibility and influence of orthopedic research, *SICOT-J* should adopt a multifaceted strategy. Promoting high-impact narrative and systematic reviews in emerging subfields can increase citation potential and readership, as such articles often synthesize evidence and guide clinical practice. Fostering international and South-South collaborations can improve research equity and diversity, particularly by engaging LMIC researchers who are often underrepresented in the orthopedic literature [14, 26]. Improving transparency in funding disclosures through stricter reporting standards can facilitate accountability and promote trust in research outputs [27]. Leveraging altmetrics and digital dissemination – such as social media activity and online attention scores – offers complementary insights into a study’s real-world influence beyond citations [28]. Special issues on emerging technologies like artificial intelligence [29–31], 3D printing [32, 33], and regenerative medicine [34] can attract innovative submissions and establish the journal as a leader in cutting-edge fields. Finally, supporting early-career researchers through mentorship, fee waivers, and educational workshops has enhanced academic development and global participation in orthopedic scholarship [35, 36].

## Conclusion

This scientometric study of *SICOT-J* (2015–2025) revealed steady growth in publication output, with 542 articles and an average of 10.29 citations per publication. Arthroplasty and trauma emerged as the dominant research themes, while review articles showed the highest citation impact. Despite global participation from 65 countries, external funding support was reported in only 5.17% of papers. These findings highlight the journal’s evolving role in global orthopedic scholarship and emphasize the need for increased funding transparency, international collaboration, and thematic diversification.

## Data Availability

The data for this paper are available in the public domain.

## Supplementary material

*Supplementary Table 1*: Distribution by Type of Documents in the *SICOT-J* (2015-2025). *Supplementary Table 2*: Subject-wise publications of *SICOT-J* (2015-2025). *Supplementary Table 3*: Top 22 Most Productive Organizations with seven or more papers. *Supplementary Table 4*: Top 19 Most Productive Authors with seven or more papers. *Supplementary Figure 1*: Published Research Areas in *SICOT-J* (2015-2025). *Supplementary Figure 2*: Countries’ Collaboration Network. *Supplementary Figure 3*: Institutional Collaboration Network. *Supplementary Figure 4*: Author’s collaboration map demonstrating the density of publications and their co-authorship connections. The supplementary material of this article is available at https://www.sicot-j.org/10.1051/sicotj/2026021/olm.
