# Supplementary material for: A decade of global orthopaedic research in SICOT-J (2015–2025): a scientometric analysis of publication trends, collaboration, and citation impact
Source: SICOT J. 2026 May 25;12:32. doi: 10.1051/sicotj/2026021 (PMC13221162; doi:10.1051/sicotj/2026021)
Supplement: Supplementary file 1 — Supplementary Table 1: Distribution by Type of Documents in the SICOT-J (2015-2025). Supplementary Table 2: Subject-wise publications of SICOT-J (2015-2025). Supplementary Table 3: Top 22 Most Productive Organizations with seven or more papers. Supplementary Table 4: Top 19 Most Productive Authors with seven or more papers. Supplementary Figure 1: Published Research Areas in SICOT-J (2015-2025). Supplementary Figure 2: Countries’ Collaboration Network. Supplementary Figure 3: Institutional Collaboration Network. Supplementary Figure 4: Author’s collaboration map demonstrating the density of publications and their co-authorship connections. [file sicotj-12-32-s1.pdf]

Supplementary Table 1: Distribution by Type of Documents in the *SICOT-J* (2015-2025)

S.No	Document Type	TP	TC	CPP	%TP
1	Research Articles	426	3200	7.51	78.60
2	Reviews	101	2361	23.38	18.63
3	Letters	5	3	0.60	0.92
4	Editorials	3	3	1.00	0.55
5	Erratum	7	10	1.43	1.29
	Total	542	5577	10.29	100.00

(TP: Total Publications; TC: Total Citations; CPP: Citations Per Publication)

Supplementary Table 2: Subject-wise publications of *SICOT-J* (2015-2025)

S.No	2015-25				TP	
	**Broad Subject Area**	**TP**	**TC**	**CPP**	**2015-2019**	**2020-2024**
1	Arthroplasty or Joint Replacement	140	1493	10.66	38	89
2	Trauma, Fracture and Dislocation	131	1049	8.01	64	63
3	Sports Injury and Arthroscopy	38	393	10.34	22	13
4	Spine/Spinal Surgery	25	175	7.00	16	8
5	Infection and Covid-19	18	248	13.78	4	13
6	Cancer/Tumor/Sarcoma	17	611	35.94	13	4
7	Pediatric Trauma & Orthopaedics	4	60	15.00	2	2
8	Regenerative Medicine	1	45	45.00	1	0

(TP: Total Publications; TC: Total Citations; CPP: Citations Per Publication)

Supplementary Table 3: Region-wise publications of *SICOT-J*

S.No	2015-2025				TP	
	**Region**	**TP**	**TC**	**CPP**	**2015-19**	**2020-24**
1	Hip	125	1228	9.82	53	65
2	Knee	89	1193	13.40	27	39
3	Spine	25	175	7.0	16	8
4	Shoulder	20	91	4.55	9	11
5	Neck	19	194	10.21	10	9
6	Elbow	11	95	8.64	8	3
7	Head	9	58	6.44	7	2
8	Ankle	4	24	6.00	1	3
9	Foot	3	18	6.00	2	1
10	Leg	3	8	2.67	1	2

(TP: Total Publications; TC: Total Citations; CPP: Citations Per Publication)

Supplementary Table 4: Top 22 Most Productive Organizations with seven or more papers

S.No	Name of the organization	TP	TC	CPP	RCI	ICP	%ICP	TLS	Collaborating links with others
**1**	Université Claude Bernard Lyon 1, France	42	435	10.36	1.01	22	52.38	327	2(38), 3(36). 4(36), 5(36), 6(32), 7(30), 8(4),
**2**	Université de Lyon, France	41	422	10.29	1.00	25	60.98	328	1(38), 4(36), 3(35), 5(33), 7(31), 6(29)
**3**	Hopital de la Croix-Rousse, France	40	427	10.68	1.04	26	65.00	297	1(36), 2(35), 4(33), 5(34), 6(33), 7(29), 8(26)
**4**	Université Gustave Eiffel, France	37	391	10.57	1.03	22	59.46	284	1(36), 2(36), 3(33), 5(33), 6(25), 8(25)
**5**	Laboratoire de Biomécanique et	36	404	11.22	1.09	23	63.89	284	1(36), 2(33), 3(34), 4(33), 6(30), 7(28),

	Mécanique des Chocs, France								8(23)
**6**	Fédération Internationale de Football Association, France	35	322	9.20	0.89	23	65.71	286	1(32), 2(29), 3(33), 4(29), 5(30), 7(27), 8(25)
**7**	Laboratoire Interuniversitaire de Biologie de la Motricité, France	33	330	10.00	0.97	23	69.70	259	1(30), 2(31), 3(29), 4(28), 5(28). 6(27), 8(19)
**8**	CHU de Lyon, France	31	307	9.90	0.96	14	45.16	249	1(27), 2(26), 3(26), 4(25), 5(23), 6(25), 7(19)
**9**	Assiut University, Egypt	24	169	7.04	0.68	12	50.00	55	
**10**	National and Kapodistrian University of Athens, Greece	19	175	9.21	0.90	10	52.63	69	1(2), 2(2), 5(2), 6(2), 8(2)
**11**	Juntendo University School of Medicine, Japan	16	162	10.13	0.98	0	0.00	13	
**12**	Ain Shams University, Egypt	14	76	5.43	0.53	3	21.43	25	
**13**	Cairo University, Egypt	12	72	6.00	0.58	5	41.67	43	
**14**	Aarhus Universitetshospital, Denmark	11	183	16.64	1.62	2	18.18	23	
**15**	Centre Hospitalier Universitaire de Nîmes, France	10	98	9.80	0.95	5	50.00	29	16(5), 22(6)
**16**	Lebanese American University Medical Center-Rizk Hospital, Lebanon	10	90	9.00	0.87	10	100.00	31	
**17**	University of Cape Town, South Africa	8	78	9.75	0.95	5	62.50	32	19(7)
**18**	Addenbrooke's Hospital,	8	126	15.75	1.53	4	50.00	38	21(5)

	Cambdidge, UK								
**19**	Groote Schuur Hospital, South Africa	7	58	8.29	0.81	5	71.43	30	17(7)
**20**	Aristotle University of Thessaloniki, Greece	7	13	1.86	0.18	4	57.14	37	3(3)
**21**	Cambridge University Hospitals NHS Foundation Trust, UK	7	52	7.43	0.72	3	42.86	33	18(5)
**22**	Université de Montpellier. France	7	62	8.86	0.86	1	14.29	19	15(6)
	Total of the top 22 organizations	455	4452	9.78	0.95	247	54.29	2791	
	Global total	542	5577	10.29	1.00				
	Share of top 22 organizations in the global total	83.95	79.83						

(TP: Total Publications; TC: Total Citations; CPP: Citations Per Publication; RCI: Relative Citation Index; ICP: International Collaborative Papers; TLS: Total Link Strength)

Supplementary Table 5: Top 19 Most Productive Authors with seven or more papers

**S. No**	**Name of the author**	**Affiliation of the author**	**TP**	**TC**	**CPP**	**RCI**	**ICP**	**%ICP**	**TLS**	**Collaborating authors**
1	S. Lustig	Hopital de la Croix-Rousse, France	42	493	11.74	1.14	28	66.67	234	2(35), 3(32), 10(9), 12(8)
2	E. Servien	Hopital de la Croix-Rousse, France	35	418	11.94	1.16	23	65.71	190	1(35), 3(29), 10(8), 12(8), 17(6)
3	C. Batailler	Hopital de la Croix-Rousse, France	32	352	11.00	1.07	20	62.50	169	1(32), 2(29), 10(8), 12(6), 17(6)
4	A.F. Mavrogenis	National and Kapodistrian University of Athens, Greece	16	170	10.63	1.03	8	50.00	106	

5	K. Kaneko	Juntendo University School of Medicine, Japan	12	157	13.08	1.27	0	0.00	13	9(9), 7(8), 16(5)
6	P. K. Kouyoumdjian	Université de Montpellier. France	11	98	8.91	0.87	5	45.45	47	11(6), 14(4)
7	T. Baba	Juntendo University School of Medicine, Japan	11	110	10.00	0.97	0	0.00	75	5(8), 9(9), 16(7)
8	H.G. Said	Assiut University, Egypt	10	81	8.10	0.79	4	40.00	28	,
9	Y. Homma	Addenbrooke's Hospital, Cambdidge, UK	10	127	12.70	1.23	0	0.00	11	7(9), 5(9), 16(5
10	E. Sappey - Marinier	Hopital de la Croix-Rousse, France	9	292	32.44	3.15	4	44.44	69	1(9), 2(8), 3(8), 12(4),
11	J. Mansour	McGill University Health Centre, Montreal, Canada	9	63	7.00	0.68	6	66.67	44	6(6). 14(4), 15(3)
12	J. Shatrov	Hopital de la Croix-Rousse, France	8	104	13.00	1.26	5	62.50	43	1(8), 2(8), 3(6), 10(4),
13	R.Coulomb	Centre Hospitalier Universitaire de Nîmes, France	8	71	8.88	0.86	2	25.00	35	1(1), 6(8), 11(5), 14(1)
14	C. Assi	Lebanese American University Medical Center-Rizk Hospital, Lebanon	8	74	9.25	0.90	8	100.00	35	1(1), 6(4), 11(4), 15(7)
15	K. Yammine	Lebanese American University Medical Center-Rizk Hospital, Lebanon	7	37	5.29	0.51	7	100.00	30	14(7), 6(3), 11(3)
16	J. Ochi	Juntendo University School of Medicine, Japan	7	83	11.86	1.15	0	0.00	50	5(5), 7(7), 9(5),
17	C.	Hopital de la Croix-	7	11	1.57	0.1	6	85.7	40	1(7), 2(7),

	Kpotserimpas	Rousse, France				5		1		3(7),
18	V. Khanduja	Addenbrooke's Hospital, Cambdidge, UK	7	102	14.57	1.42	4	57.14	12	
19	M. Ishijima	Addenbrooke's Hospital, Cambdidge, UK	7	32	4.57	0.44	0	0.00	51	5(4), , 7(3), 9(2)
		Total of top 19 authors	256	2875	11.23	1.09	130	50.78	1282	
		Global total	542	5577	10.29	1.00				
		Share of top 19 authors in global total	47.23	51.55						

(TP: Total Publications; TC: Total Citations; CPP: Citations Per Publication; RCI: Relative Citation Index; ICP: International Collaborative Papers; TLS: Total Link Strength)

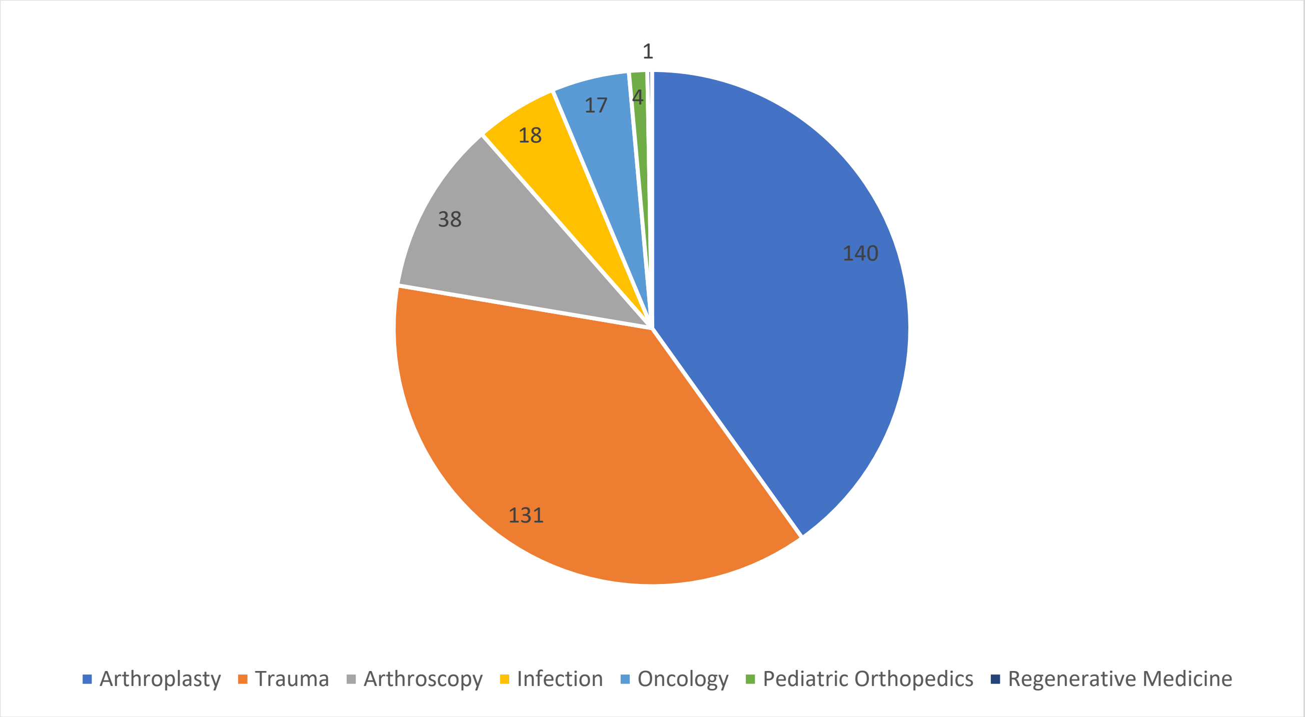

Supplementary Figure 1: Published Research Areas in *SICOT-J* (2015-2025)

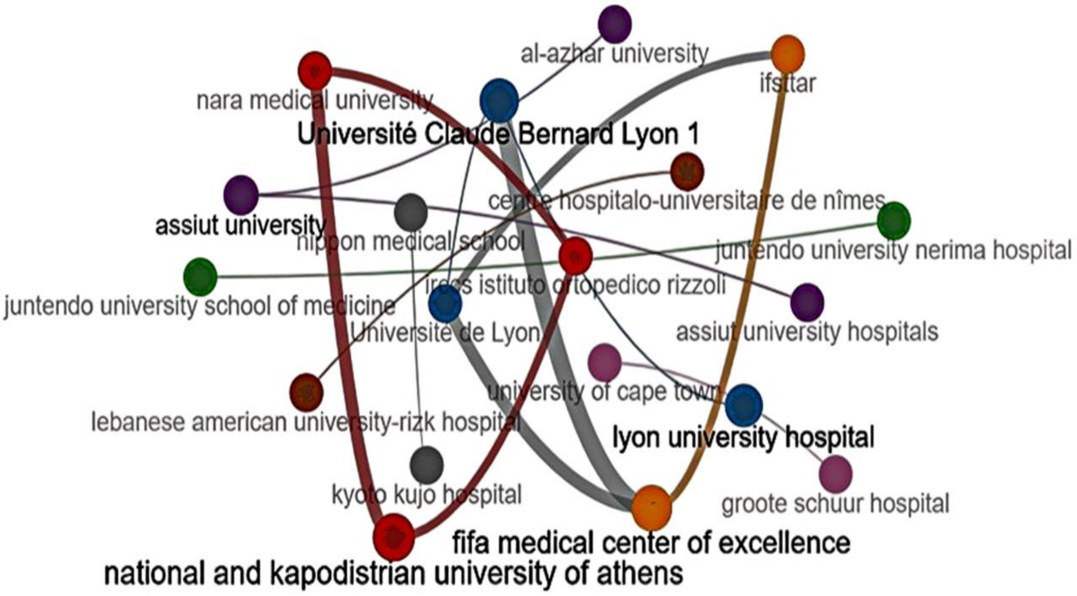

Supplementary Figure 2: Institutional Collaboration Network

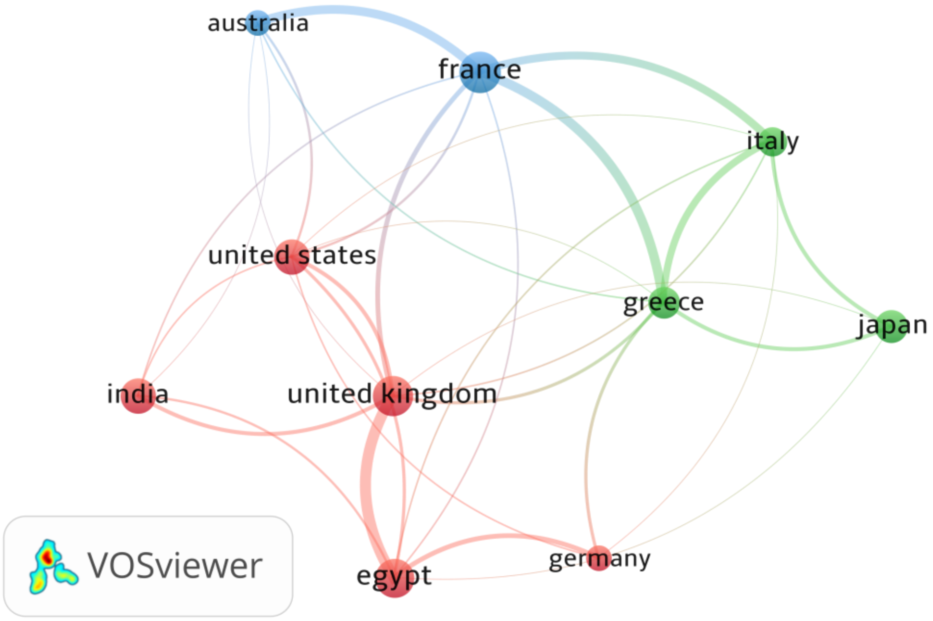

Supplementary Figure 3: Countries’ Collaboration Network

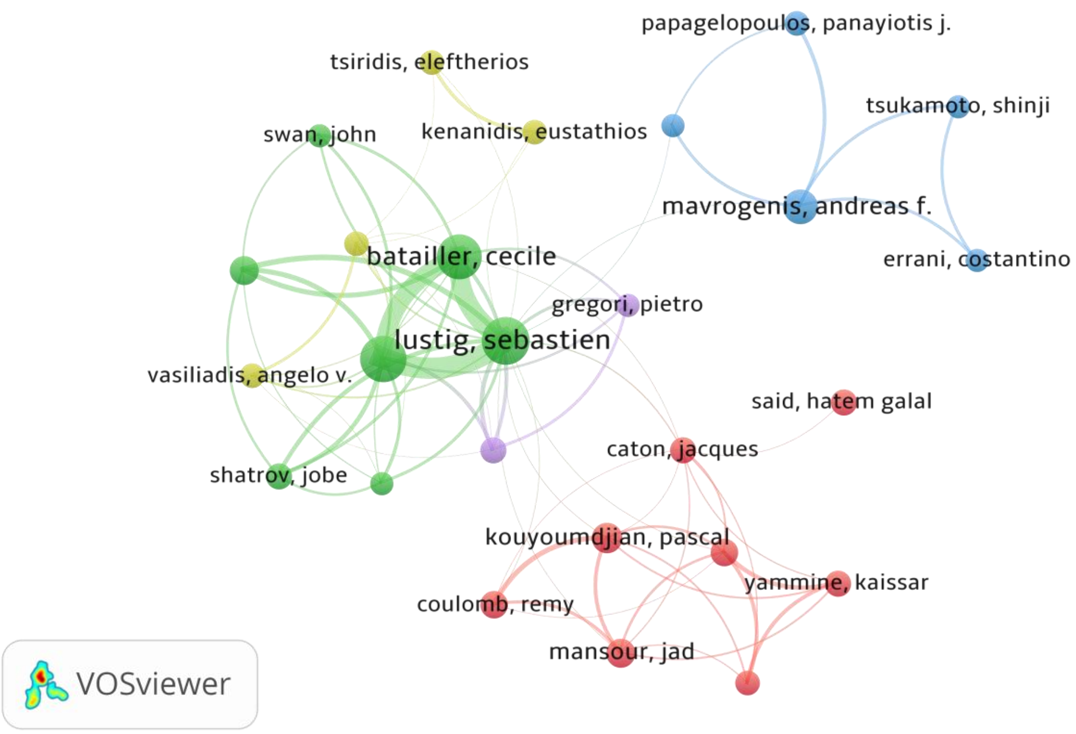

Supplementary Figure 4: Author's collaboration map demonstrating the density of publications and their co-authorship connections

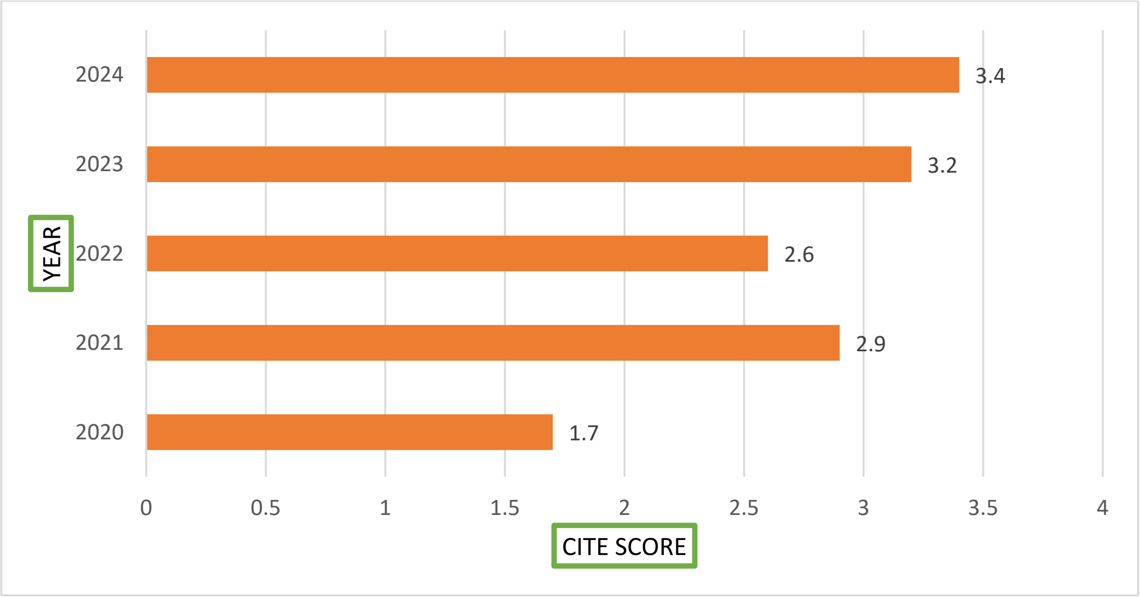

Supplementary Figure 5: Cite Score Trends of *SICOT- J* (2015-2024)
